# The Influence of Grain Interactions on the Plastic Stability of Heterophase Interfaces

**DOI:** 10.3390/ma7010302

**Published:** 2014-01-13

**Authors:** Jason R. Mayeur, Irene J. Beyerlein, Curt A. Bronkhorst, Hashem M. Mourad

**Affiliations:** Theoretical Division, Los Alamos National Laboratory, Los Alamos, NM 87545, USA; E-Mails: irene@lanl.gov (I.J.B.); cabronk@lanl.gov (C.A.B.); hmourad@lanl.gov (H.M.M.).

**Keywords:** crystal plasticity, rolling texture, nanolamellar composites, interface stability, grain interactions

## Abstract

Two-phase bimetal composites contain both grain boundaries and bi-phase interfaces between dissimilar crystals. In this work, we use a crystal plasticity finite element framework to explore the effects of grain boundary interactions on the plastic stability of bi-phase interfaces. We show that neighboring grain interactions do not significantly alter interface plastic stability during plane strain compression. The important implications are that stable orientations at bimetal interfaces can be different than those within the bulk layers. This finding provides insight into bi-phase microstructural development and suggests a pathway for tuning interface properties via severe plastic deformation.

## 1. Introduction

Bulk bimetal nanocomposites are generating much interest due to their high strength and hardness [1–10]. Recent experiments show that these bimetal nanocomposites, in contrast to bulk single phase nanocrystalline metals, are surprisingly stable in extreme conditions, such as high temperature, shock, high temperature deformation, severe plastic deformation, and radiation [1,4,11–14]. Unlike traditional composites, these nanomaterials contain a high density of interfaces, spaced nanometers apart. In addition to being strong obstacles to dislocation motion the bimetal interfaces are clearly playing a key role in defect ‘management’; however, very little is known about the physical properties of the interfaces themselves. Only very recently have atomic-scale studies revealed how deformation mechanisms, such as slip transmission, plastic slip and deformation twinning, are affected by interface structure [15–17].

Bulk bimetal nanocomposites can be fabricated by severe plastic deformation (SPD) techniques and the interface characteristics evolve as a function of the imposed deformation [1,2]. Bimetal interfaces within nanolayered composites fabricated by accumulative roll bonding (ARB) are found to stabilize to a particular interface type [18,19]. If the ARB processing pathway is changed, then another stable predominant interface prevails [20]. Both of these interfaces are distinct from those that develop in nanolayered composites fabricated by physical vapor deposition (PVD) [18]. In the case of PVD composites, the dominant interface character is driven by low formation energy. However, in SPD composites *plastic stability* also plays a role in the emergence of the predominant interface. Plastic stability describes the stability of the bimetal interface character (five-parameter description of a boundary) as a function of plastic deformation. Thus, to be plastically stable, both crystals comprising an interface must be able to slip without reorienting. The stability of crystal orientations for various monophase fcc and bcc single and polycrystalline metals have been well characterized experimentally and investigated using crystal plasticity models [21–26]. In contrast, much less is known about the plastic stability of bi-phase interfaces. Developing an understanding of the plastic stability of such interfaces is critical for designing processing methodologies for bulk bimetal nanocomposites.

Crystals attached to bi-phase interfaces have different microstructural ‘neighborhoods’ and this can affect its plastic stability. For example, ARB fabrication begins with a stack of polycrystalline sheets, each of which contain multiple grains through the thickness. As the composite is rolled, the individual layer thickness decreases as does the number of grains spanning each layer. At some critical layer thickness, only a single grain spans a given layer [10]. With further deformation, the average grain aspect ratio increases [19], such that each grain is primarily bounded by two bimetal interfaces. Thus, for composites that contain multiple grains through the thickness, interface grains are surrounded by neighboring grains of the same phase within each layer as well as grains of dissimilar phase along the interface, whereas each grain in the fine layered composites (single grain through the thickness) is primarily bounded by grains of the dissimilar phase. Therefore, it is expected that the plastic stability of the interface could vary as a function of layer thicknesses due to the potential influence or lack thereof grain interactions within individual layers.

In this work, we use a bi-phase crystal plasticity finite element model to explore the competition between grain boundary interactions and heterophase interactions on the plastic stability of a bimetal interface. To compare different interface configurations, we develop stability parameters based on the influence of interface misorientation and misorientation rates with strain. The results show that the bimetal interface constraints have a profound effect on the plastic stability. The implications of this study are that in plastically deformed bimetal composites, the grains adjoining interfaces have different orientation distributions than those within the layers. These results help to explain experimental observations of texture gradients reported in multilayered composites deformed in rolling [20]. The results of our computational study also indicate that when the layers refine to single crystalline layers via plastic deformation, the texture is primarily governed by the interface.

## 2. Stability of Rolling Textures

Before moving forward, it is instructive to briefly review prior work on the stability of grains as single crystals, embedded in a polycrystalline, or surrounded by bimetal interfaces. The evolution of stable orientations in rolling has been extensively studied for single phase materials. A stable orientation or orientation distribution (texture) is classified as one in which the crystal orientations do not substantially change with increasing strain. Experimentally and computationally, stable orientations can be characterized by non-decreasing change in intensity and/or volume fraction as a function of deformation [23,25,27]. These studies have underscored the importance of a number of factors that affect the texture evolution such as the imposed strain level, initial texture, grain morphology, and the slip system level hardening behavior. They found that these factors influence the inter- and intragranular slip system activity both in terms of the number of active (or dominant) systems and the relative proportions of accumulated slip on those systems, which directly drives crystal reorientation.

## 3. Stability of Layered Bi-Phase Composites in Rolling

Recently crystal plasticity formulations have been employed to study multilayers Cu-Nb, Cu-Ni, Zr-Nb, and Cu-Ag composites under deformation [4,28-34]. Anderson *et al.* [28] and Al-fadhalah *et al.* [29] studied Cu-Nb multilayered composites produced by PVD, which contain thermodynamically stable fcc-bcc interfaces, e.g., Kurdjumov-Sachs (KS) ND || 〈111〉_fcc_ || 〈101〉_bcc_ and II 〈110〉_fcc_ || 〈111〉_bcc_ in-plane and Nishiyama-Wasserman (NW) ND || 〈111〉_fcc_ || 〈110〉_bcc_ and 〈110〉_fcc_ || 〈001〉_bcc_ in-plane interfaces [35]. Using a composite grain model within a viscoplastic self-consistent (VPSC) scheme wherein all Cu and Nb crystals were initially joined with KS orientation relationship with 〈111〉_fcc_|| 〈110〉_bcc_ || ND and random in-plane orientations of the composite grains with respect to the RD, Al-fadhalah *et al.* [29] showed the KS interface was unstable and that the expected rolling texture characteristic of each phase developed. A separate set of simulations was performed with the constraint that the KS OR be maintained during rolling, and the results compared favorably with the experimentally measured textures of samples with 75 nm layer thickness. This result suggests that the texture evolution in these materials is intimately tied to the preservation of the KS OR, and it has been argued that the KS OR is maintained due to the symmetric activation of slip systems in the individual layers. Anderson *et al.* [28] used a crystal plasticity model based on the principle of minimum shear to study the stability of Cu and Nb single crystals with [111]_fcc_ || [110]_bcc_ || ND under rolling conditions in an effort to understand the development of the atypical rolling textures in PVD Cu-Nb. By systematically varying the initial in-plane orientations (perturbations of each crystal by a rotation about the ND), they were able to identify several mutually stable orientations as well as predict the primary texture component that was experimentally observed.

More recently, plastic stability studies have been carried out in which the interface characters were allowed to evolve [30–33,36]. In Knezevic *et al.* [33], the VPSC scheme was used to evaluate texture development of the Zr phase in multilayered ARB Zr-Nb composites with micron layer thickness. The number of grains through the thickness of each layer was so large that interfaces had little effect on texture evolution in the Zr phase. In [30], crystal plasticity finite element (CPFE) modeling was employed to study texture development in ARB Cu-Nb composites with layers that were several microns thick. That study demonstrated that the bi-phase CPFE model was able to reproduce classical rolling textures for each phase that are observed for ARB Cu-Nb composites with the layer thickness >1 μm. In both studies, the possibility of texture gradients due to interface effects were not studied computationally or experimentally.

In another study by Mayeur *et al*. [31], the two-phase CPFE method was applied to examine the intrinsic stability (*i.e.*, in the absence of grain interaction effects) of a bicrystal during rolling. The bicrystal configuration was a periodic cell containing a single interface character, and thus models a multilayered composite containing single crystalline layers. Thus, grain interaction effects on plastic stability were not considered. That work revealed that the plastic stability of the interface is essentially dictated by the least stable of the two crystal orientations comprising the interface. These results helped explain the emergence of special interface types observed in nanolayered composites, which were associated with texture components that deviated from those seen in the composites with layer thicknesses on the order of microns. This model was also used to explain the differences observed between the strengths of ARB and PVD Cu-Nb composites in elevated temperature deformation [4].

Very recently, experiments on ARB composites with layers several microns thick pointed to a through-thickness texture gradient [20]. Understanding how such texture gradients evolved during the rolling process requires understanding the competition between interface interactions and grain-grain interactions on the plastic stability of grains at the interface. This is an issue that has not been previously studied. To this end, in the present work we use the bi-phase CPFE framework used in Hansen et al. [30] and the bicrystal model of Mayeur *et al*. [31] to study plastic stability of bi-phase interfaces under rolling. Herein, simulations are performed for different grain and interface configurations to systematically elucidate the effect of nearest neighbor grain interactions and its relative contribution to the plastic stability of interface character.

## 4. Crystal Plasticity Finite Element Modeling

**Figure 1.**
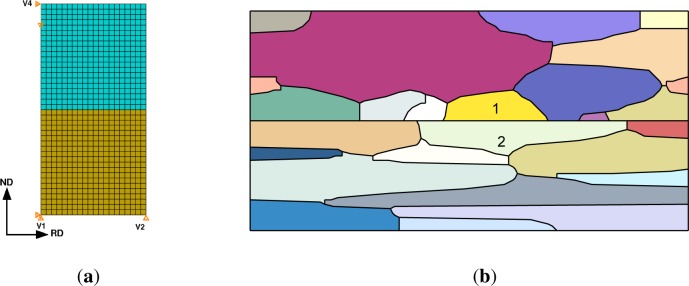
Finite eleement models: (**a**) periodic; (**b**) multicrystal grain morphology.

The crystal plasticity constitutive model is used to study two microstructural configurations as shown in Figure 1. The first configuration is the ‘multicrystal’ model, which consists of two multicrystalline layers, one of Cu and the other Nb. There are multiple grains along the interface of each layer and the interface character (orientation relationship and interface plane) of one pair of joined Cu and Nb grains is given a specific relationship. The remaining grains are assigned orientations sampled from the measured texture of each phase [30]. The second configuration is the bicrystal model used by Mayeur *et al*. [31], which does not account for nearest neighbor grain interactions. The bicrystal model is employed to study the same set of interfaces in order to elucidate the role of neighbor grain interactions on plastic stability. The specific interfaces studied in the present work (discussed in Section 5) are chosen because they are experimentally observed in fine scale Cu-Nb multilayered composites, e.g., either the ARB Cu-Nb composites with submicron layer thickness or the PVD Cu-Nb composites.

### 4.1. Constitutive Model

The constitutive model [37] used to describe the response of the Cu and Nb phases is a classical local finite deformation formulation of single crystal plasticity based on a two-term multiplicative decomposition of the deformation gradient into elastic, F*^e^*, and plastic, F*^p^*, parts, *i.e*.,

$$F=F^{e}F^{p}$$ (1)

Dislocation motion, which leaves lattice vectors unaltered, is encapsulated by F*^p^* and the stretching and rotating of the lattice is given by F*^e^*. The evolution equation for F*^p^* is defined in terms of the plastic velocity gradient with respect to the intermediate configuration as:

$${\bar{L}}^{p}={\dot{F}}^{p}F^{p}{}^{{}^{-1}}=\underset{\alpha }{\sum }{\dot{\gamma }}^{\alpha }{\bar{s}}^{\alpha }⊗{\bar{n}}^{\alpha }$$ (2)

where ${\dot{\gamma }}^{\alpha }$ is the slip system shearing rate, ${\bar{s}}^{\alpha }$ is the slip vector, and ${\bar{n}}^{\alpha }$ is the slip plane normal. Since the lattice vectors are assumed to be unaltered by dislocation glide, the lattice slip vector, $s^{\alpha }$, and the slip plane normal, $n^{\alpha }$, are given in the current configuration as:

$$s^{\alpha }=F^{e}{\bar{s}}^{\alpha },\;n^{\alpha }=F^{e-T}{\bar{n}}^{\alpha }$$ (3)

Equation (3) provides the means to track the evolution of the crystallographic orientations of the crystal, which are embedded in the rotational part of F*^e^* that can be obtained via the polar decomposition.

The kinetic equation for the slip system shearing rate is based on thermally-activated dislocation motion, *i.e*.,

$${\dot{\gamma }}^{\alpha }={\dot{\gamma }}_{0}\text{exp}\left[-\frac{F_{0}}{\kappa \theta }{\langle 1-{\langle \frac{|\tau^{\alpha }|-s^{\alpha }\frac{\mu }{\mu_{0}}}{s^{\alpha }\frac{\mu }{\mu_{0}}}\rangle }^{p}\rangle }^{q}\right]\text{sgn}(\tau^{\alpha })$$ (4)

where ${\dot{\gamma }}_{0}$ is the reference shearing rate, *F*_0_ is the activation free energy, *k* is Boltzmann’s constant, *θ* is the absolute temperature, *μ* is the shear modulus, *μ*_0_ is the shear modulus at 0 K, *τ_α_*is the resolved shear stress, is the slip resistance,*S^α^* is the intrinsic lattice resistance, $s_{l}^{\alpha }$ and *p* and *q* are exponents that characterize the shape of the obstacle profile. The slip resistance is governed by the following equations

$${\dot{s}}^{\alpha }=\sum_{\beta }h^{\alpha \beta }|{\dot{\gamma }}^{\beta }|,\;s^{\alpha }(t=0)=s_{0}$$ (5)

$$h^{\alpha \beta }=\left[r+(1-r)\delta_{\alpha \beta }\right]h^{\beta }$$ (6)

$$h^{\beta }=h_{0}\left(\frac{s_{s}^{\beta }-s^{\beta }}{s_{s}^{\beta }-s_{0}^{\beta }}\right)$$ (7)

$$s_{s}^{\beta }=s_{s_{0}}{\left(\frac{{\dot{\gamma }}^{\beta }}{{\dot{\gamma }}_{0}}\right)}^{\frac{k\theta }{A}}$$ (8)

where *S*_*S*0_ is the initial slip resistance, *h^αβ^* is the hardening interaction matrix, *r* is the latent hardening ratio, *h*_0_ is the initial hardening modulus, $S_{S}^{\beta }$ is the saturation slip resistance, and *S*_*S*0_ and *A* are material parameters that control the rate and temperature dependence of the saturated value of the slip resistance.

In prior work, the stability of individual fcc and bcc orientations have been found to depend on the choice of hardening parameters used in phenomenological hardening laws, such as rate sensitivity parameters, latent hardening coefficients, or in the particular case of bcc metals, the ratio of critical resolved shear stresses of the {110}111〉 slip to {112}〈111〉 slip systems [21–23,25,27]. In our simulations, we consider the {111}〈110 slip systems for Cu and the {110}〈111 and {112}〈111〉 slip systems for Nb. Including both {110} and {112} slip systems is critical for capturing the plastic anisotropy of bcc metals as recently demonstrated by Wang and Beyerlein [38] in their discrete dislocation dynamics study. The constitutive parameters in the flow rule and hardening relations have been determined by fitting the single phase Cu and Nb responses to experimental data over a wide range of strain rates and temperatures. The model calibration was performed by Hansen *et al.* [30] and the material parameters are listed in Appendix (Table 2).

### 4.2. Bicrystal Model

The bicrystal model, shown in Figure 1a, consists of a Nb crystal joined to a Cu crystal with the interface normal in the *x*_2_-direction (ND), subjected to plane strain compression boundary conditions. Periodicity of the displacements is enforced [39] in both the *x*_1_ and *x*_2_ directions and the boundary conditions at the control vertices are illustrated in the schematic. In the simulations the Nb crystal is stacked on top of the Cu crystal, however, the stacking sequence is not important due to the periodicity constraints. Displacements are fully constrained at vertex 1, the *x*_2_ displacement component is constrained at vertex 2, and the *x*_1_ component of displacement is fixed at vertex 3. Loading is applied at vertex 3 via displacement control at a constant strain rate of 0.08 s^−1^.

### 4.3. Multicrystal Model

The grain morphology for the multicrystal finite element model is shown in Figure 1b. The primary grains of interest that have the prescribed interface OR are located in the center of the volume element and are denoted by labels “1” and “2”, where grain 1 is in the Cu layer and grain 2 is in the Nb layer. Five different multicrystal realizations were considered for each interface. Each realization has a different orientation distribution for the grains surrounding the prescribed interface grains. The grain morphology and orientation distributions were constructed to be consistent with measurements obtained for an as-rolled ARB Cu-Nb composite material with 24 μm layer thickness as described by Hansen *et al*. [30]. The microstructural volume element has dimensions of 50 μm × 100 μm and contains 26 grains (14 Cu and 12 Nb). The grains are meshed using 6-noded triangular hybrid elements (CPE6H). The rolling process is modeled as plane strain compression with isochoric boundary conditions that are enforced via multipoint constraints. The rolling direction is parallel to the *x*_1_-axis and the normal direction is parallel the *x*_2_-axis. The microstructural statistical volume element was subjected to 50% rolling reduction at a strain rate of 0.1 s^−1^.

## 5. Results

The interfaces studied are summarized in Table 1. Each orientation is expressed in the rolling plane-rolling direction convention, where {*hkl*} is the rolling plane and 〈*uvw*〉 the rolling direction. The“dashed” shorthand notation for the interface lists the orientation (or component) for the Cu crystal before the dash and then the orientation for the Nb crystal after the dash. For example, the C-I interface refers to the C orientation of the Cu crystal and the I orientation of the Nb crystal. KS is short for Kurdjumov-Sachs (see Section 3), the interface characteristic of PVD Cu-Nb multilayer composites. The configurations listed in Table 1 are representative of interfaces that according to the prior calculations are intrinsically metastable (C-I, D-I), stable (Brass-I,Goss-I), and unstable (KS) in rolling.

### 5.1. Plastic Stability Metrics

To compare the plastic stability of the different interfaces, we consider two primary variables: the magnitude of the lattice rotation and its rate of change with respect to the deformation history. In particular, we are interested in how these quantities are influenced by grain interactions. The results are discussed in terms of what can be considered measures of the overall and instantaneous plastic stability of the interface configurations at 50% reduction. The bicrystal simulations serve to establish the baseline (intrinsic) plastic stability of the interfaces to which the multicrystal simulation results will be compared.

**Figure 2.**
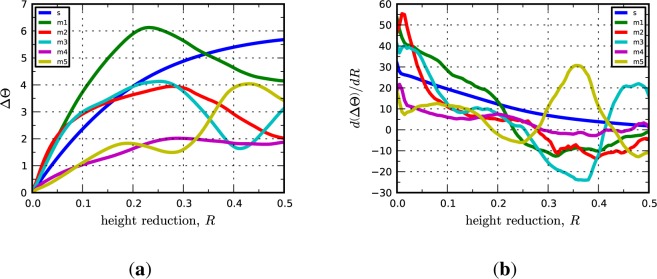
Lattice rotation (**a**) angle; and (**b**) rate plotted as functions of height reduction for the Cu phase in the C-I interface. In the legend, “s” refers to the periodic bicrystal simulations and the “m1, m2, …” refer to the different realizations of the multicrystal simulations.

The total plastic stability is defined in terms of the magnitude of lattice rotation, ∆Θ, in each phase due to the applied deformation, and the angle is determined from the elastic (lattice) rotation tensor, **R***^e^*, as:

$$\Delta \Theta =\text{arccos}\left(\frac{{\text{tr}R}^{e}-1}{2}\right)$$ (9)

where **R***^e^* is the orthogonal rotation tensor associated with the polar decomposition of F*^e^*. The rotation angles are computed at the integration point level and then averaged over the grain to give a single value of ∆Θ. It is also informative to characterize the instantaneous stability of a crystal orientation as a function of the applied deformation. The lattice spin rate, **W***^e^*, is defined as:

$$W^{e}=\text{skw}(L-L^{p})$$ (10)

where **L** and **L***^p^* are total and plastic velocity gradients, respectively, defined on the current configuration. In the following, we take a simpler approach to quantifying the rate of lattice reorientation in terms of only the rotation angle. The lattice spin rate tensor, **W***^e^*, contains information about both the angle and axis of lattice rotation and we found metrics based on the norm of **W***^e^* to be too noisy for our purposes. Instead, we use the rate of change of ∆Θ with respect the height reduction, *R*, as a metric of instantaneous lattice reorientation. The rates, $\frac{d\Delta \Theta }{dR}$, are computed numerically from plots of the average value of ∆Θ as a function of *R*. Figure 2 shows representative plots of ∆Θ *vs.R* and $\frac{d\Delta \Theta }{dR}$ *vs. R*.

### 5.2. C-I and D-I Interfaces

The common interfaces in ARB composites lie between the C-I and D-I interfaces in orientation space. We first examine these interfaces using the bicrystal simulations with no grain-grain interaction effects. These simulations show that neither the ideal C-I or D-I interface is perfectly stable. Both interfaces reorient slightly under the applied rolling deformation. At 50% reduction the Cu crystal in the C-I interface rotates 5.7° about the minus TD (towards the D component) to a component the lies between the C and D components and the Nb crystal rotates 2.04°, also about the minus TD. In contrast, the Cu crystal in the D-I interface rotates in the opposite direction (towards the C component) by an angle of 3.8° whereas the Nb crystal rotates nominally about the minus TD by 5.0°. It is interesting to note that, while the Cu crystal in each interface is rotating toward the other component, e.g., the C component is rotating toward the D component and vice versa, they do not stabilize at the same final orientation. Perhaps more surprisingly, there is some overshoot. Here overshoot means that the Cu crystal initially in the C component orientation reoriented to a position in orientation space beyond the final position of the crystal initially in the D component orientation. However, this overshoot is minor at 50% reduction and the final orientations of the Cu crystals in each case are only separated by 1.5°. The final orientations are relatively stable in that the rates of lattice rotation, as measured by $\frac{d(\Delta \Theta )}{dR}$, are 0.11° and 3.99° for the Cu and Nb phases, respectively, in the C-I interface and 5.46° and 7.69° for the Cu and Nb phases, respectively, in the D-I interface. Although, the interface plane and OR are strictly lost, the C-I and D-I interfaces are classified as metastable since the crystal orientations have stabilized in close proximity to the initial configurations. Notably, the prediction that the stable interfaces lie between C-I and D-I is in agreement with experimental measurements [18,19].

**Figure 3.**
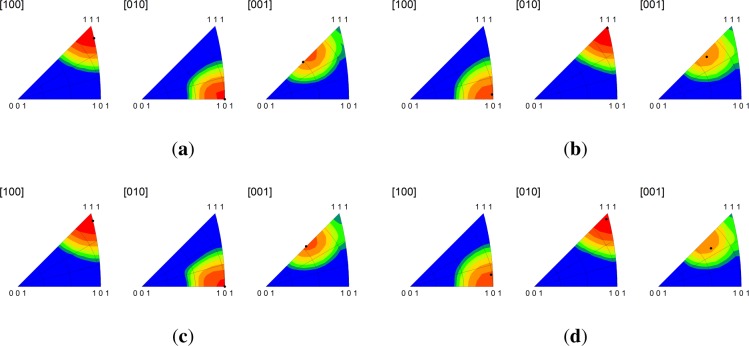
Combined inverse pole figures at 50% reduction from the multicrystal simulations for the (**a**) copper phase in the C-I interface; (**b**) niobium phase in the C-I interface; (**c**) copper phase in the D-I interface; and (**d**) niobium phase in the D-I interface. The final orientations obtained from the periodic bicrystal simulations are indicated by the filled black circles. RD[100]/TD[010]/ND[001].

**Figure 4.**
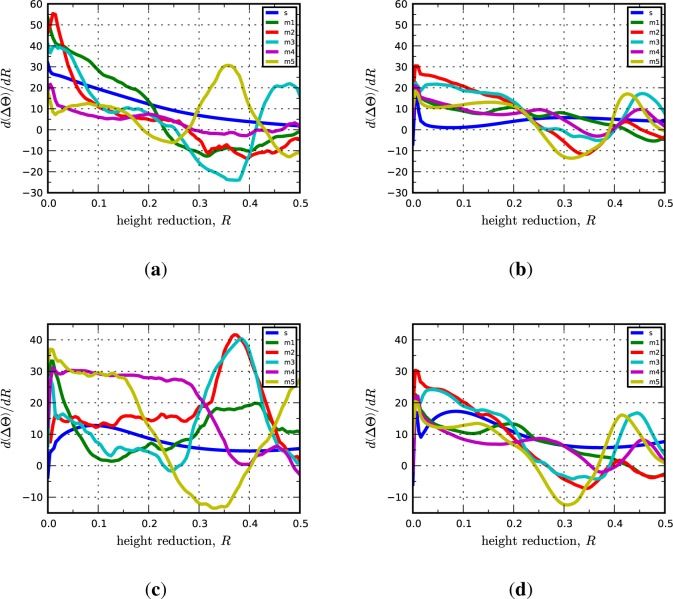
Lattice reorientation rate plotted as a function of height reduction for the (**a**) copper phase in the C-I interface; (**b**) niobium phase in the C-I interface; (**c**) copper phase in the D-I interface; and (**d**) niobium phase in the D-I interface. In the legend, “s” refers to the periodic bicrystal simulations and the “m1, m2, …” refer to the different realizations of the multicrystal simulations.

Significantly, we find that the multicrystal simulation results are consistent with the periodic bicrystal results for both the C-I and D-I interfaces. The combined inverse pole figures (IPFs) for each microstructural realization (m1-m5) for the C-I and D-I interfaces are given in Figure 3 where the final orientations from the periodic bicrystal simulations are indicated in the contour plots with filled black circles. The average misorientation angles calculated from the multicrystal simulations are actually smaller for the Cu phase in the C-I interface (2.91°) and for the Nb phase in the D-I interface (3.68°) as compared to what is observed in the periodic bicrystal simulations. This result is somewhat surprising since it is expected that the additional heterogeneity introduced by grain interactions would serve to decrease the plastic stability. The average lattice rotations for the Nb phase in the C-I interface (3.56°) and the Cu phase in the D-I interface (7.4°) display the anticipated trend in that the total lattice rotations have been amplified due to the deformation heterogeneity. The lattice rotation rates plotted as a function of height reduction are given in Figure 4 for both interfaces. As shown, the multicrystal rotation rates display more pronounced oscillatory behavior compared to the periodic bicrystal simulation results. Notably, this plot also reveals the that the multicrystalline response curves appear to be approximating and/or oscillating about the asymptotic response of the periodic bicrystal simulations.

### 5.3. Goss-I and Z-I Interfaces

**Figure 5.**
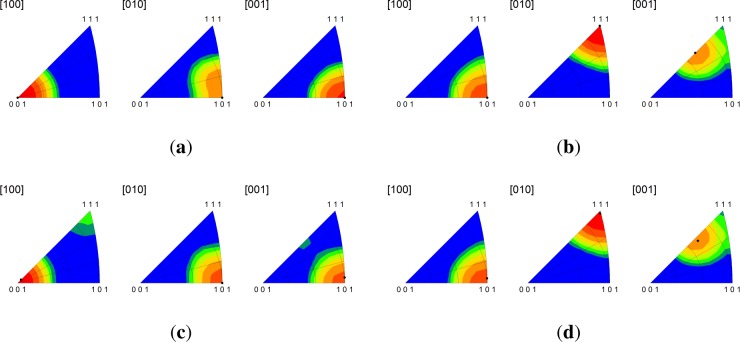
Combined inverse pole figures at 50% reduction from the multicrystal simulations for the (**a**) copper phase in the Goss-I interface; (**b**) niobium phase in the Goss-I interface; (**c**) copper phase in the Z-I interface; and (**d**) niobium phase in the Z-I interface. The final orientations obtained from the periodic bicrystal simulations are indicated by the filled black circles. RD[100]/TD[010]/ND[001].

The Goss-I and Z-I interfaces (see Table 1) are experimentally observed in very fine layered ARB composites [40]. The bicrystal simulations reveal that the Goss-I interface is extraordinarily stable under plane strain compression as lattice rotations in each phase are less than 1°, whereas the Z-I interface is unstable. In fact, the Cu crystal in the Z-I interface rotates 10.17° about the minus TD to a near Goss orientation, while the Nb crystal is stable and rotates 1.89° about the TD. Due to the Goss-I interfaces exceptional stability, the rates of lattice reorientation in both crystals of the Goss-I interface at 50% reduction are negligible. The results also demonstrate that the final orientations of the Z-I interface, which correspond to a near Goss-I interface, are also stable as indicated by the small lattice reorientation rates of 5.37° and 3.55° for Cu and Nb, respectively. To assess the influence of grain interactions, we compare in Figure 5 the inverse pole figures for the Goss-I and Z-I interfaces in the bicrystal and multicrystal configurations. The results show that the final orientations predicted by the multicrystal calculations are consistent with the bicrystal calculations for both interfaces.

**Figure 6.**
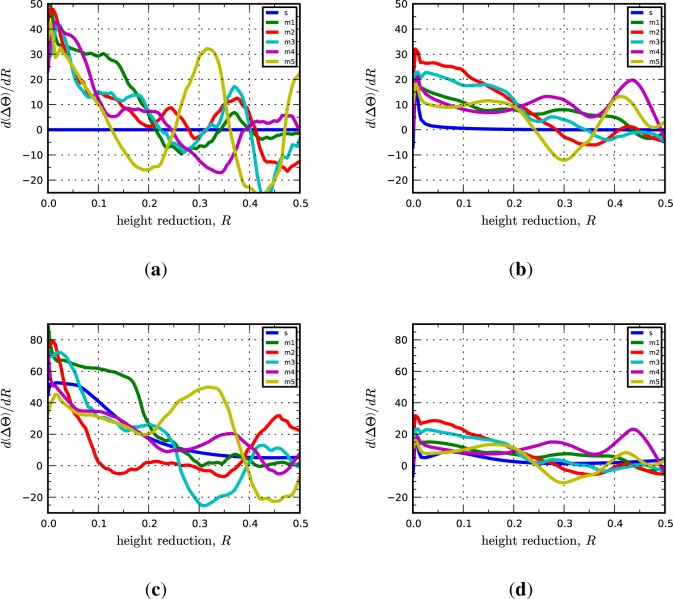
Lattice reorientation rate plotted as a function of height reduction for the (**a**) copper phase in the Goss-I interface; (**b**) niobium phase in the Goss-I interface; (**c**) copper phase in the Z-I interface; and (**d**) niobium phase in the Z-I interface. In the legend, “s” refers to the periodic bicrystal simulations and the “m1, m2, …” refer to the different realizations of the multicrystal simulations.

In the multicrystal calculations, the Goss-I interface remains stable although magnitude of the lattice reorientation in each phase has slightly increased from <1° to average values over the five realizations of 3.21° and 3.85° for Cu and Nb, respectively. As in the bicrystal simulation, the Z-I interface reorients primarily to a Goss-I interface although the IPFs indicate there is a small amount of the {101}〈111〉 component in the Cu phase. The average lattice rotation in the Cu phase of the Z-I interface is roughly the same as in the bicrystal simulation (9.76°), while it has increased slightly in the Nb phase to 3.89°. As in the previous set of results and shown in Figure 6, the evolution of the lattice rotation rates in the multicrystal simulations oscillate about the bicrystal response curves. Based on these calculations, we conclude that the plastic stability of the Goss-I and Z-I interfaces is not affected by grain interactions.

### 5.4. The Brass-I Interface

**Figure 7.**
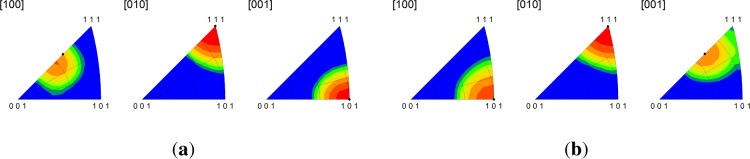
Combined inverse pole figures at 50% reduction from the multicrystal simulations for the (**a**) copper phase in the Brass-I interface; and (**b**) niobium phase in the Brass-I interface. The final orientations obtained from the periodic bicrystal simulations are indicated by the filled black circles. RD[100]/TD[010]/ND[001].

**Figure 8.**
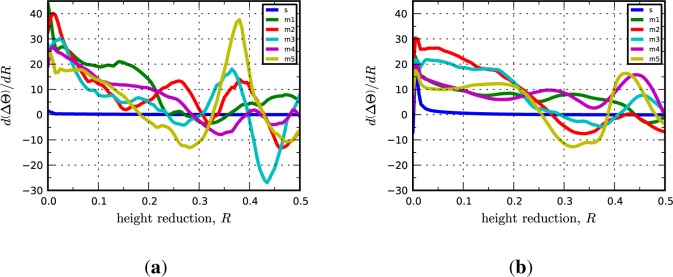
Lattice reorientation rate plotted as a function of height reduction for the (**a**) copper phase in the Brass-I interface; and (**b**) niobium phase in the Brass-I interface. In the legend, “s” refers to the periodic bicrystal simulations and the “m1, m2, …” refer to the different realizations of the multicrystal simulations.

The Brass-I interface is observed at lower frequencies in Cu-Nb ARB composites as compared to the C-I and D-I interfaces; however, texture measurements suggest that it is present for all submicron to nanolayer thicknesses [10,19]. The Brass-I interface is predicted to be remarkably stable by the bicrystal simulations. Both crystals rotate less than 1° from their original orientation and the lattice reorientation rates are essentially zero throughout the duration of loading. Similarly, the multicrystal simulations reveal that the Brass-I interface is stable given that the average lattice rotations at 50% reduction over the five microstructural realizations are 3.47° and 3.72° for Cu and Nb, respectively. The only notable difference is revealed by the inverse pole figures shown in Figure 7, which indicate that the peak direction aligned along the RD in the Cu phase has shifted from the 〈112〉 direction to a direction in the vicinity of 〈338〉. No shift is observed in the IPFs of the Nb phase. The evolution of the lattice rotation rates given in Figure 8 are qualitatively similar to those for the previously considered interfaces. As before, we find that the plastic stability of the Brass-I interface is not significantly affected by grain interactions.

### 5.5. The KS Interface

Thus far we have examined interfaces that appear in multilayered Cu-Nb composites manufactured by ARB. As previously mentioned, the KS interface is predominant in PVD Cu-Nb composites but is not observed in ARB Cu-Nb composites [18]. In the bicrystal simulation, the KS interface is shown to be highly unstable. At 50% reduction, the Cu crystal has rotated by 25.91° about the TD and the Nb crystal by 27.63° about the minus TD such that a 53° misorientation has developed between the two crystals with respect to the initial orientations. As might be expected, the lattice reorientation rates for both phases at 50% reduction are significantly higher than those observed for the ARB “x”-I interfaces-19.28° and 36.62°, for Cu and Nb, respectively. This result suggests that the lattice orientations have not stabilized at 50% reduction.

The multicrystal simulations, consistent with the bicrystal results, show that the KS interface is highly unstable. It is noteworthy that the average lattice rotations computed for the multicrystal calculations are approximately the same as those in the bicrystal calculations. In other words, the intrinsic instability of the interface is not further amplified by grain interaction effects, at least in terms of its average behavior. The KS interface is also unique from the “x”-I interfaces studied previously in that grain interaction effects lead to substantially different final orientations as compared to those obtained with the bicrystal model. As indicated by the IPFs, the Cu phase in the bicrystal simulations has reoriented to a texture distributed about the D component and the Nb phase to the I component. In contrast, the Cu phase develops two components in the multicrystal simulation: one is near the predicted bicrystal orientation, but closer to the C orientation, and the other is the Goss component. The Nb phase in the multicrystal calculations is not affected by this additional reorientation behavior in Cu and continues to exhibit a strong I component orientation. Similar to the other interfaces studied, the lattice rotation rate response curves oscillate about the bicrystal response curves for the respective phases. However, in contrast to the other interfaces, the lattice reorientation at 50% reduction does not appear to have stabilized, although one might expect that texture saturation is imminent since the phases have reoriented to stable rolling orientations and/or stable interface configurations, *i.e*., C-I and Goss-I.

## 6. Discussion

The interfaces studied in the present work were motivated by experimental characterization of the ARB and PVD Cu-Nb lamellar composites. This characterization has shown that single crystal-like textures develop in ARB composites when the layer thicknesses are submicron [10,18,41]. Further, two distinct classes of predominant interfaces develop: (1) the “submicron interface”, which lies in the vicinity of the C-I and D-I type of interfaces and is the primary interface prior to the onset of extensive twinning in the Cu phase and (2) the “nano interface”, which is near the Goss-I and Z-I interfaces and becomes the dominant interface after substantial reorientation of the Cu phase due to twinning when the layer thicknesses are < 50 nm [40]. Prior to the onset of twinning, the Goss-I interface is also observed, but not as frequently as the near C-I/D-I interface.

**Figure 9.**
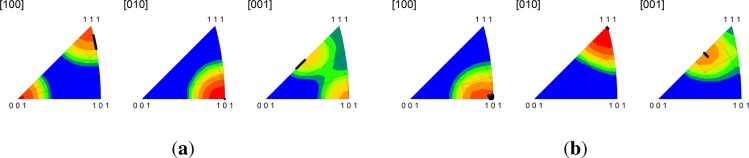
Combined inverse pole figures at 50% reduction from the multicrystal simulations for the (**a**) copper phase in the KS interface; and (**b**) niobium phase in the KS interface. The final orientations obtained from the periodic bicrystal simulations are indicated by the filled black circles. RD[100]/TD[010]/ND[001].

Additionally, we studied the Brass-I interface, which is a secondary type of “submicron interface” in the ARB composites and the KS interface, which is the predominant interface of PVD Cu-Nb lamellar composites. In general, the periodic bicrystal and multicrystal calculations predict similar final orientations for the grains of interest at 50% reduction for the interfaces considered. The orientations from the multicrystal calculations (given by the IPF contour plots) essentially represent distributions centered about the predictions of the bicrystal results. The notable exception is the Cu crystal in the KS interface. As shown in Figure 9a, a Goss component of texture develops in the Cu phase in addition to the near {112}〈111〉 component that is predicted by the bicrystal simulations. This result is significant because it shows that the KS interface reorients under rolling to form two interfaces, the C-I and Goss-I interfaces, which according to our calculations are stable and also prominent in the Cu-Nb lamellar composites.

Mayeur *et al.* [31] demonstrated that under the kinematic constraint of co-deformation the intrinsic plastic stability (no grain interactions) during rolling is determined by the less plastically stable orientation of the two making up the interface. To quantify the plastic stability of an interface, a simple metric based on the average angle of lattice rotation in each crystal was introduced. The interface stability parameter, *Ω*, is defined as:

$$\Omega =\text{exp}\left(\frac{\Delta \Theta_{\text{Cu}}}{\epsilon }\right)\text{exp}\left(-\frac{\Delta \Theta_{\text{Nb}}}{\epsilon }\right)$$ (11)

where and are the average angles of the lattice rotation within the Cu and Nb crystals, respectively, at a given strain level є. є is the magnitude of the true compressive strain and is given in terms of the rolling reduction, *R*, as

ϵ=|1n(1−R)| (12)

**Figure 11.**
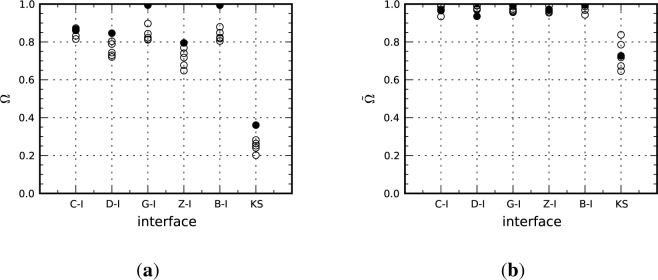
Plastic stability parameters plotted for each of the considered interfaces (**a**) total stability; and (**b**) instantaneous stability at 50% reduction. Filled circles are from the periodic bicrystal simulations and open circles are for the different multicrystal realizations.

Additionally, we introduce a rate form of Equation (11) as a measure of the instantaneous plastic stability of the interface at 50% reduction denoted as $\tilde{\Omega }$. The instantaneous stability parameter, is defined as in Equation (11) except the rotation angle and strain are replaced with their respective rates. The interface stability parameters for each of the interfaces studied are given in Figure 11 where the filled circles represent the periodic bicrystal result and the open circles represent the results for the five different orientation distributions from the multicrystal simulations.

The comparisons made in Figure 11 indicate that grain interactions reduce the total plastic stability in general. The lone exception is the C-I interface, which actually exhibits slightly improved stability for two of the microstructural realizations, although the improvement is modest. Interestingly, the metastable interfaces, i.e., those with 0.8 < *Ω <* 0.9, appear to be less sensitive to extrinsic influences than interfaces that have high (Goss-I, Brass-I) intrinsic stability. This result is expected since one would not anticipate the near-perfect stability to be retained in the presence of heterogeneous deformations induced by grain interactions. It is also interesting to note that the plastic stability of the C-I interface is comparable to that of the Goss-I and Brass-I interfaces when grain interactions are considered. As shown in Figure 11b, all of the “x”-I interfaces have very high values of the instantaneous plastic stability parameter, which implies that orientations given by the IPFs for these interfaces have more or less stabilized at 50% reduction. Furthermore, there is much less scatter in the $\tilde{\Omega }$ plot between the bicrystal and multicrystal calculations for these interfaces as compared to the Ω plot, a result that suggests that extrinsic affects have a more pronounced effect on the magnitude of the lattice rotation than on the rate at which these interfaces establish a stable orientation. The instantaneous interface stability parameter plot also shows that the KS interface has not stabilized at 50% reduction and suggests that the C-I and Goss-I interface textures will continue to sharpen with increased deformation.

The simulation results support the experimental observation that the preferred “submicron interface” lies between the C and D components in the Cu phase and few degrees from the I component in the Nb phase. These interfaces are shown to have moderately high total intrinsic plastic stability that is not substantially degraded by the extrinsic grain interactions. It is also shown that the Goss-I and Brass-I interfaces, which have near perfect total intrinsic stability parameter values, are quite stable in the multicrystal simulations as well; a result that is also in accord with experimental characterization of ARB Cu-Nb composites. According to the simulations, the Z-I and KS interfaces are unstable and reorient to achieve more favorable texture components. The Z-I interface moves toward a Goss-I interface and the KS interface evolves into a mix of C-I and Goss-I interfaces. The simulations indicate that the Z-I interface observed in the 10 nm ARB composite by Zheng *et al*. [40] is not plastically stable, wherein the Z component in Cu would diminish at the expense of the Goss component if the nanocomposite were rolled further. The KS interface results agree with the observation of Misra *et al*. [35] that the interface is unstable at 50% reduction for PVD Cu-Nb multilayered composites with *h* = 75 μm.

Recently, it has been proposed that the emergence of predominant interfaces in ARB Cu-Nb multilayer composites can be explained through a combination of two key properties: (1) high mechanical stability under rolling deformation (plastic stability) and (2) low interface formation energy [1]. Due to the large amount of heterophase interfacial area that is created during the ARB process, the texture evolution will be biased toward interfaces that have low formation energy. Beyerlein et al. [1] have used these criteria to explain why the predominant “submicron” and “nano” interfaces emerge at the expense of other stable single phase rolling components.

## 7. Conclusions

This work presents a computational study of the plastic stability of Cu-Nb heterophase interfaces in rolling and the effects of neighbor grain interactions. The study focuses on interfaces that emerge in ARB Cu-Nb multilayer composites after extreme straining (the layer thickness below 1 μm). For the six interfaces studied, the simulations show that the final orientations predicted by the multicrystal simulations with neighbor grain interactions are similar to those predicted from a bicrystal simulation with no neighbor grain interactions. In most cases, the bicrystal model yields a uniform reorientation field within each crystal, whereas the multicrystal model predicts a heterogeneous field, which is essentially distributed about the average reorientation from the bicrystal calculation. On this basis, we conclude that co-deformation constraints imposed by the bimetal interfaces are strong, while those by neighboring grain boundaries are relatively weak. As a consequence, the orientational stability conditions of grains at interfaces are altered and thus, different texture components can be expected at interfaces than within the bulk. These findings can help explain the texture gradients observed in coarse-grained multilayered composites and why special preferred orientation distributions emerge at interfaces in nano-grained multilayered composites.

## Figures and Tables

**Figure 10.**
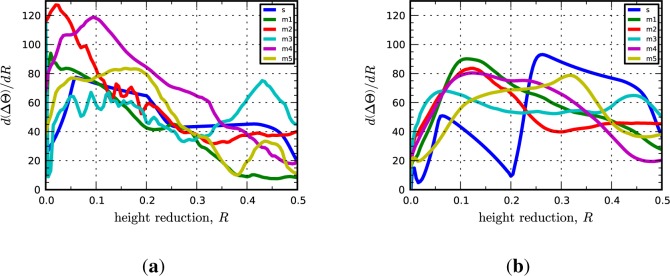
Lattice reorientation rate plotted as a function of height reduction for the (**a**) copper phase in the KS interface; and (**b**) niobium phase in the KS interface. In the legend, “s” refers to the periodic bicrystal simulations and the “m1, m2, …” refer to the different realizations of the multicrystal simulations.

**Table 1.** Interface Oreintation Relationships (ND) [RD].

Interface	Cu	Nb
C-I	$\left(\bar{1}21)\;[11\bar{1}\right]$	$\left(1\bar{2}\bar{1})\;[101\right]$
D-I	$\left(\bar{4}\bar{4}11\right)$[11118]	$\left(1\bar{2}\bar{1})\;[101\right]$
Gloss-I	(101)[010]	$\left(1\bar{2}\bar{1})\;[101\right]$
Z-I	$\left(51\bar{5})\;[11\bar{0}\bar{1}\right]$	$\left(1\bar{2}\bar{1})\;[101\right]$
Brass-I	(101) $\left[\bar{1}21\right]$	$\left(1\bar{2}\bar{1})\;[101\right]$
KS	$\left(11\bar{1})\;[1\bar{2}\bar{1}\right]$	(101) $\left[\bar{1}21\right]$

## Appendix

Calibrated constitutive parameters for the single crystal models.

**Table 2.** Constitutive model parameters.

Parameter	Cu	Nb	Units
*ρ*	8960	8400	kg m^−3^
*c_p_*	380	268	J kg^−1^ K^−1^
*α*	17.7	8.7	*μ*m m^−1^ K^−1^
*η*	0	0	–
*m*_11_	−36.3	−17.7	MPa K^−1^
*C*_110_	179.5	242.0	GPa
*m*_12_	−16.4	1.92	MPa K^−1^
*C*_120_	126.4	121.2	GPa
*m*_44_	−25.7	2.18	MPa K^−1^
*C*_440_	82.5	28.0	GPa
*r*	1.4	1.4	–
${\dot{\gamma }}_{0}$	10^7^	10^7^	s^−1^
*s*_0_	1	40	MPa
*s_l_*	20	700	MPa
*F_0_*	1.0**×**10^−18^	2.1×10^−19^	J
*p*	0.33	0.34	–
*q*	1.66	1.66	–
*S*_S0_	205	140	MPa
*h*_0_	200	300	MPa
*A*	1.5**×**10^−19^	1.0**×**10^−17^	J
